# The Impact of *Saccharomyces cerevisiae* on a Wine Yeast Consortium in Natural and Inoculated Fermentations

**DOI:** 10.3389/fmicb.2017.01988

**Published:** 2017-10-16

**Authors:** Bahareh Bagheri, Florian F. Bauer, Mathabatha E. Setati

**Affiliations:** Department of Viticulture and Oenology, Institute for Wine Biotechnology, Stellenbosch University, Stellenbosch, South Africa

**Keywords:** yeast consortium, population dynamics, yeast interactions, wine fermentation, ARISA

## Abstract

Natural, also referred to as spontaneous wine fermentations, are carried out by the native microbiota of the grape juice, without inoculation of selected, industrially produced yeast or bacterial strains. Such fermentations are commonly initiated by non-*Saccharomyces* yeast species that numerically dominate the must. Community composition and numerical dominance of species vary significantly between individual musts, but *Saccharomyces cerevisiae* will in most cases dominate the late stages of the fermentation and complete the process. Nevertheless, non-*Saccharomyces* species contribute significantly, positively or negatively, to the character and quality of the final product. The contribution is species and strain dependent and will depend on each species or strain’s absolute and relative contribution to total metabolically active biomass, and will therefore, be a function of its relative fitness within the microbial ecosystem. However, the population dynamics of multispecies fermentations are not well understood. Consequently, the oenological potential of the microbiome in any given grape must, can currently not be evaluated or predicted. To better characterize the rules that govern the complex wine microbial ecosystem, a model yeast consortium comprising eight species commonly encountered in South African grape musts and an ARISA based method to monitor their dynamics were developed and validated. The dynamics of these species were evaluated in synthetic must in the presence or absence of *S. cerevisiae* using direct viable counts and ARISA. The data show that *S. cerevisiae* specifically suppresses certain species while appearing to favor the persistence of other species. Growth dynamics in Chenin blanc grape must fermentation was monitored only through viable counts. The interactions observed in the synthetic must, were upheld in the natural must fermentations, suggesting the broad applicability of the observed ecosystem dynamics. Importantly, the presence of indigenous yeast populations did not appear to affect the broad interaction patterns between the consortium species. The data show that the wine ecosystem is characterized by both mutually supportive and inhibitory species. The current study presents a first step in the development of a model to predict the oenological potential of any given wine mycobiome.

## Introduction

The alcoholic fermentation of grape must, whether inoculated or not with commercial starter cultures, is initiated by a complex yeast community comprising a high proportion of oxidative and weakly fermentative yeasts (Jolly et al., 2003a; Ghosh et al., 2015; Wang et al., 2015). These species are rapidly outgrown by strongly fermentative yeasts that dominate the middle and end of fermentation (Pretorius et al., 1999; Jolly et al., 2003b; Zott et al., 2008; Bagheri et al., 2015; Ghosh et al., 2015; Setati et al., 2015; Wang et al., 2015; Morgan, 2016; Portillo et al., 2016; Tristezza et al., 2016). The growth and metabolic activity of these yeast species are influenced by physicochemical conditions that prevail during the fermentation process including the rapid depletion of nutrients and oxygen and the accumulation of ethanol (Sainz et al., 2003; Mendoza et al., 2009). However, beyond such environmental or chemical factors, ecological interactions between yeast species will primarily determine the wine fermentation dynamics and the outcome of the fermentation process (Nissen and Arneborg, 2003; Pina et al., 2004; Sadoudi et al., 2012; Renault et al., 2013; Morales et al., 2015; Wang et al., 2015; Shekhawat et al., 2017). For many years, research evaluated interactions between strains of *S. cerevisiae*, the main wine fermenting yeast, with a focus on killer toxin-producing strains (Branco et al., 2014; Williams et al., 2015; Albergaria and Arneborg, 2016; Pérez-Torrado et al., 2017). However, with the growing interest in non-*Saccharomyces* yeast species and the commercialization of a few species for use as co-inoculants in controlled mixed starter fermentations, attention has turned toward evaluating yeast–yeast interactions holistically (Ciani and Comitini, 2015; Albergaria and Arneborg, 2016; Ciani et al., 2016; Wang et al., 2016). Undoubtedly, wine microbial consortia are difficult to scrutinize. Consequently, some studies have employed simplified models in which the interaction between two species mainly *S. cerevisiae* and non-*Saccharomyces* species were investigated (Andorra et al., 2011; Wang et al., 2014; Englezos et al., 2015; Shekhawat et al., 2017). Several aspects, including inoculum ratio, the timing of inoculation of *S. cerevisiae*, cell-cell contact and production of inhibitory metabolites, have been investigated in order to decipher the mechanisms underlying yeast–yeast interactions during wine fermentation (Gobbi et al., 2013; Branco et al., 2014, 2015; Izquierdo Cañas et al., 2014; Kemsawad et al., 2015; Lencioni et al., 2016). Despite these efforts, the overall interactions among wine yeast species in a fermentation modulated by multiple species remain unclear.

Synthetic microbial consortia composed of a subset of culturable strains that simulate the natural community and preserve the indigenous interactions shaped by co-adaptation/evolution, provide a tractable model system with reduced complexity (De Roy et al., 2014; Ponomarova and Patil, 2015), which makes it easier to study interspecific interactions (Jagmann and Philipp, 2014; Jiang et al., 2017). Such a model system also opens opportunities to employ methods inapplicable to complex systems, e.g., species quantitation can easily be done with selective plating, quantitative PCR, fluorescent *in situ* hybridization (FISH), and flow cytometry (Xufre et al., 2006; Grube and Berg, 2009; Zott et al., 2010; Ponomarova and Patil, 2015). These methods have been applied successfully to monitor population dynamics in wine fermentation. However, they are not without limitations. For instance, FISH and qPCR, require species-specific probes and primers whereas, flow cytometry requires prior knowledge of initial microbial population in order to label different species (Deere et al., 1998; Malacrinò et al., 2001; Prakitchaiwattana et al., 2004; Hierro et al., 2006a; Xufre et al., 2006; Andorrà et al., 2010a,b; Zott et al., 2010). In contrast, Automated Ribosomal Intergenic Spacer Analysis (ARISA), which mainly relies on the heterogeneity of the ITS1-5.8S rRNA-ITS2 gene, has been used successfully in several ecological studies (Brežná et al., 2010; Kraková et al., 2012; Ghosh et al., 2015). Like other methods, ARISA may also introduce bias since it is unable to differentiate live and dead cells. However, ARISA is an efficient and rapid tool that can provide a snapshot of the population dynamics (Hierro et al., 2006a; Ramette, 2009; Brežná et al., 2010; Kraková et al., 2012; O’Sullivan et al., 2013; Cangelosi and Meschke, 2014; Ženišová et al., 2014; Ghosh et al., 2015).

The current study aimed to evaluate the application of a multi-species yeast consortium as a tool to investigate population dynamics and yeast–yeast interactions in wine fermentation. The constructed model consortium resembles natural wine yeast consortia in so far as comprising species with different fermentative capacities (i.e., weakly fermentative, medium fermentation capacity and strongly fermentative). Moreover, the consortium was formulated based on species that have been encountered and found in sometimes dominant numbers in grape musts from different South African wine regions and cultivars (Jolly et al., 2003a; Weightman, 2014; Bagheri et al., 2015; Ghosh et al., 2015; Morgan, 2016). The model consortium was evaluated in synthetic must in the presence and absence of *S. cerevisiae*, as well as in a real grape juice that differed significantly from the synthetic must. To allow for a rapid and accurate monitoring of the population dynamics, ARISA was optimized and assessed for its suitability and reliability as a tool to semi-quantitatively monitor yeast dynamics in the model consortium.

The data show that *S. cerevisiae* strongly and specifically suppresses certain non-*Saccharomyces* yeast species, while also favoring the persistence of other species. The findings suggest that the complex modulation of the yeast ecosystem by *S. cerevisiae* will influence the outcome of wine fermentation by selectively changing the contribution of non-*Saccharomyces* species.

## Materials and Methods

### Yeast Strains and Culture Conditions

Sixteen yeast isolates obtained from the culture collection of the Institute for Wine Biotechnology (IWBT) and two commercial yeast species, *S. cerevisiae* Lalvin EC1118 (Lallemand, Canada) and *Torulaspora delbrueckii* BIODIVA (Lallemand, Canada) were used in this study (**Table 1**). The yeast stock cultures were maintained in 20% (v/v) glycerol at -80°C and were streaked out on Wallerstein Laboratory Nutrient agar (WLN) (Sigma–Aldrich, Spain) when required. The plates were incubated at 30°C for 3–5 days.

**Table 1:** Strains used in this study and their ITS1-5.8S rRNA-ITS2 gene sizes.

Species	Strains number	ITS Size (bp)
*Hanseniaspora uvarum (Hu)*	Y1104	747
*Hanseniaspora vineae (Hv)*	Y980	740
*Hanseniaspora opuntiae* (*Ho*)	Y866	748
*Pichia terricola (Pt)*	Y974	419
*Issatchenkia orientalis (Io)*	Y1130	490
*Starmerella bacillaris* (*Sb*)	Y975	458
*Candida apicola* (*Cap)*	Y957	457
*Candida azyma (Ca)*	Y979	436
*Candida parapsilosis (Cp*)	Y842	522
*Candida glabrata (Cg)*	Y800	884
*Torulaspora delbrueckii (Td)*	BIODIVA	797
*Rhodotorula glutinis (Rg)*	Y824	614
*Rhodosporidium diobovatum (Rd)*	Y840	618
*Kazachstania aerobia (Ka)*	Y845	751
*Lachancea thermotolerans* (*Lt*)	Y973	675
*Saccharomyces cerevisiae (Sc)*	EC1118	842
*Wickerhamomyces anomalus (Wa)*	Y934	618
*Metschnikowia pulcherrima (Mp)*	Y981	377

### Automated Ribosomal Intergenic Spacer Analysis (ARISA)

Single colonies of each yeast species were inoculated into 5 mL YPD broth (10 g/L yeast extract, 20 g/L peptone and, 20 g/L glucose) and incubated for 16 h at 30°C. Two milliliters of cultures were centrifuged at 5630 × *g* for 10 min to collect the cells. Genomic DNA was extracted using the method described by Sambrook and Russell (2006). DNA concentration was determined spectrophotometrically, using the NanoDrop^®^ND-1000 (NanoDrop Technologies Inc., Wilmington, DE, United States). The ITS1-5.8S rRNA-ITS2 gene was amplified using the carboxy-fluorescein labeled ITS1 primer (5′-6-FAM- TCC GTA GGT GAA CCT TGC GG-3′) and ITS4 (5′- TCC GTA GGT GAA CCTTGC GG-3′) in a 25 μL reaction, containing 50 ng DNA, 1U Takara Ex Taq, DNA polymerase (TaKaRa Bio Inc., Olsu, Shiga, Japan), 1 × Taq buffer, 0.25 μM of each primer, 400 μM dNTP mix and 1 mM MgCl_2_. The PCR reaction was performed under the following conditions: an initial denaturation of 3 min at 94°C, followed by 40 cycles of denaturation at 94°C for 30 s, annealing at 54°C for 30 s, extension at 72°C for 45 s and a final extension step of 72°C for 10 min (Slabbert et al., 2010). Three independent PCR reactions were performed. The PCR products were excised from the gel and purified using the Zymoclean^TM^ Gel DNA Recovery Kit Short Protocol (Zymo Research Corporation, Irvine, CA, United States). The ARISA fragments were separated by capillary electrophoresis at the Stellenbosch University Central Analytical Facility on an ABI 3010x Genetic Analyzer (Applied Biosystems) with a ROX 1.1 labeled size standard (75-1121 base pairs). ARISA profiles were analyzed using Genemapper software version 4.1 (Applied Biosystems). Only fragments with peak area larger than 0.5% of the total fluorescence were considered for further analysis. A bin size of 3 bp for species with ITS region below 700 and 5 bp for species with ITS region above 700 bp, was employed to minimize the inaccuracies in the ARISA analysis (Slabbert et al., 2010). The relative abundance of each peak was calculated by dividing individual peak area with the total peak areas for the respective sample.

### Micro-Fermentations

#### Fermentation in Synthetic Grape Must

Eight yeast species *viz. Metschnikowia pulcherrima, Pichia terricola, Starmerella bacillaris, Candida parapsilosis, Wickerhamomyces anomalus, Lachancea thermotolerans, Hanseniaspora vineae*, and *S. cerevisiae* were selected to establish a consortium based on (i) their frequent occurrence in grape juices from SA and other wine producing regions, (ii) easy and consistent resolution in ARISA, and (iii) easy morphological detection on WL agar (Jolly et al., 2003a; Combina et al., 2005; Di Maro et al., 2007; Lopandic et al., 2008; Romancino et al., 2008; Salinas et al., 2009; Sun et al., 2009; Suzzi et al., 2012; Weightman, 2014; Maturano et al., 2015; Morgan, 2016). Fermentations were carried out, by inoculating the selected yeast species, in synthetic grape juice medium (pH 3.5) adapted from Bely et al. (1990) and Henschke and Jiranek (1993). The medium contained 200 g/L sugars (100 g/L glucose and 100 g/L fructose) and 300 mg/L assimilable nitrogen (460 mg/L NH_4_Cl and 180 mg/L amino acids). Five hundred milliliters of the juice was dispensed into 500 mL Erlenmeyer flasks, fitted with CO_2_ traps. The juice was inoculated with the *NS-Sc* (non-*Saccharomyces-Saccharomyces*) consortium comprising of 7 non-*Saccharomyces* yeast species (*M. pulcherrima, P. terricola, S. bacillaris, C. parapsilosis, W. anomalus, L. thermotolerans*, and *H. vineae*), each inoculated at 10^6^ cells/mL and *S. cerevisiae* at 10^3^ cells/mL, and the *NS* (non-*Saccharomyces*) consortium which only consisted of the seven non-*Saccharomyces* yeasts. The fermentations were performed at 25°C with no agitation. Fermentations were monitored by weighing the flasks regularly to measure CO_2_ loss. Furthermore, samples were collected regularly to determine sugar concentrations using Fourier Transform Infra-Red Spectroscopy on the Foss Wine scan 2000 (Rhine Ruhr, Denmark). Samples were withdrawn at 2-day intervals and yeast population dynamics was monitored by direct plating on WLN agar and ARISA.

### Real Must Fermentation

Fifty liters of clarified Chenin blanc grape juice was obtained from a commercial cellar. The chemical composition of juice was measured, using spectroscopy technique by Foss wine scan 2000 (Rhine Ruhr, Denmark). The yeast community composition of the juice was determined by serial dilution and direct plating on WL-agar, followed by identification through ITS-5.8S rRNA amplification, RFLP, and sequencing as described in Bagheri et al. (2015). Subsequently, 1.5 L Chenin blanc grape juice was dispensed into 2 L fermentation bottles. Three sets of fermentations were performed: (i) spontaneous (ii) *Sc*-inoculated fermentation (at 10^3^ cells/mL, *S. cerevisiae* EC1118), and (iii) *NS-Sc* consortium inoculated (7 non-*Saccharomyces* at 10^6^ cells/mL vs. *S. cerevisiae* at 10^3^ cells/mL). The fermentations were performed in triplicate, at 25°C, and without SO_2_ addition. The fermentations were weighed daily to monitor CO_2_ release and samples were withdrawn at 2-day intervals to monitor population dynamics. The residual sugar at the end of fermentation was measured. The fermentations were considered complete when residual sugars in wine were less than 2 g/L and the yeast population dynamics was monitored by direct plating on WLN agar.

### Statistical Analysis

The DNA extraction, ARISA analysis, and fermentations were performed in triplicate. The values were presented as means ± SD. The differences between treatments were determined using analysis of variance (ANOVA) with the statistical software Statistica version 13.0 (StatSoft Inc., Tulsa, OK, United States). The differences were considered significant should the *p*-values were equal or less than 0.05. For multivariate data analysis, the Principal Component Analysis was performed, using XLSTAT in Microsoft^®^Excel (2016).

## Results

### Selection of Yeast Species for the Consortium

**FIGURE 1:**
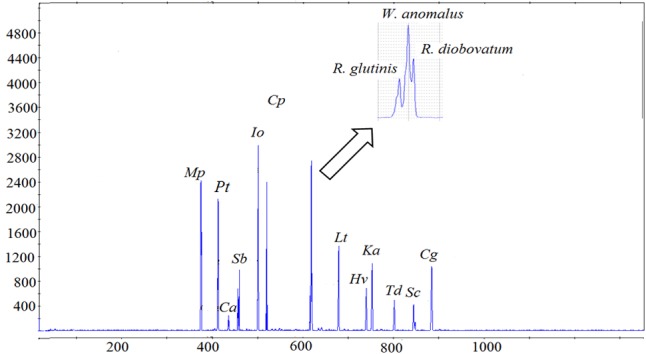
Electropherogram of a mixed culture of 18 yeast species, generated via PCR amplification with ITS1F-ITS4 primers. The *x*-axis represents the fragment size (bp) and the *y*-axis represents the relative fluorescence intensity. The following abbreviations were used for names of yeast species. *Mp, Metschnikowia pulcherrima; Pt, Pichia terricola; Ca, Candida azyma; Sb, Starmerella bacillaris; Io, Issatchenkia orientalis; Cp, Candida parapsilosis; Lt, Lachancea thermotolerans; Hv, Hanseniaspora vineae; Ka, Kazachstania aerobia; Td, Torulaspora delbrueckii; Sc, Saccharomyces cerevisiae; Cg, Candida glabrata.*

**FIGURE 2:**
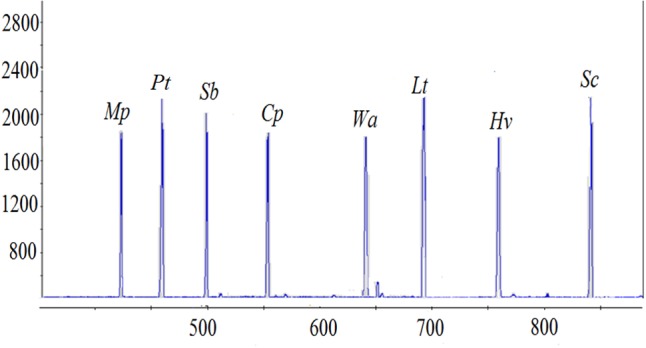
Quantitative validation between the ARISA peaks of eight selected yeast species and CFU/mL. All yeast species were inoculated at 10^5^ CFU/mL. The *x*-axis represents the fragment size (bp) and the *y*-axis represents the relative fluorescence intensity.

Eighteen yeast species commonly isolated from South African grape musts (Jolly et al., 2003a; Weightman, 2014; Bagheri et al., 2015; Morgan, 2016), were initially evaluated for DNA extractability and resolvability in ARISA analysis. The ARISA profile of the mixed community only revealed 13 peaks (**Figure 1**). An overlap between *Rhodotorula glutinis* (614 bp), *R. diobovatum* (618 bp) and *W. anomalus* (618 bp) was observed. Similarly, *H. uvarum* (747 bp), *H. opuntiae* (748 bp), and *Kazachstania aerobia* (751 bp), as well as *S. bacillaris* (458 bp) and *C. apicola* (458 bp) co-migrated and could not be resolved. Consequently, eight species (*M. pulcherrima, P. terricola, S. bacillaris, C. parapsilosis, W. anomalus, L. thermotolerans, H. vineae*, and *S. cerevisiae*), which could be reliably resolved in ARISA, and could be distinguished based on their colony morphology on WLN agar, were selected to establish a model consortium. The efficiency of DNA extraction method and ARISA on the consortium was evaluated. In addition, standard curves of optical density (OD_600__nm_) vs. colony forming units (CFU/mL) were established for each species (data not shown). A cell suspension containing approximately each at 10^5^ CFU/mL was prepared. Total genomic DNA was extracted from the mixed culture and ARISA was performed. Similar peak heights and peak areas were observed for all species, suggesting that the DNA extraction method and ARISA were efficient for all of them (**Figure 2**).

### Validation of ARISA in the Model Consortium

The detection limit of ARISA was investigated in different inoculation scenarios, representing low and high levels of selected yeast species (**Table 2**). The data indicated that when all species were inoculated at the same level, they could be detected even at 10^3^ CFU/mL while, in a situation where one species was significantly higher in concentration (≥10^6^ CFU/mL), other species could be detected if present at 10^4^ CFU/mL but not at 10^3^ CFU/mL (Supplementary Figure S1). Therefore, the detection limit of ARISA was defined as the lowest cell concentration (10^4^ CFU/mL) that resulted in a positive signal and fluorescence intensity above 50 relative fluorescence units (RFU).

**Table 2:** Yeast inoculum combinations used to determine ARISA detection limits.

Yeast species	A	B	C
*H. vineae*	10^3^	10^4^	10^3^
*S. bacillaris*	10^3^	10^4^	10^3^
*C. parapsilosis*	10^3^	10^4^	10^3^
*P. terricola*	10^3^	10^4^	10^3^
*L. thermotolerans*	10^3^	10^6^	10^3^
*W. anomalus*	10^3^	10^4^	10^3^
*M. pulcherrima*	10^3^	10^4^	10^3^
*S. cerevisiae*	10^3^	10^4^	10^6^

To test the repeatability and reliability of ARISA for monitoring the yeast dynamics throughout the fermentation, three independent DNA extractions were performed from a sample in which the yeasts were mixed in different concentrations. In each case, similar peak profiles were observed for triplicates with minor variations in peak intensities (Supplementary Figure S2).

**FIGURE 3:**
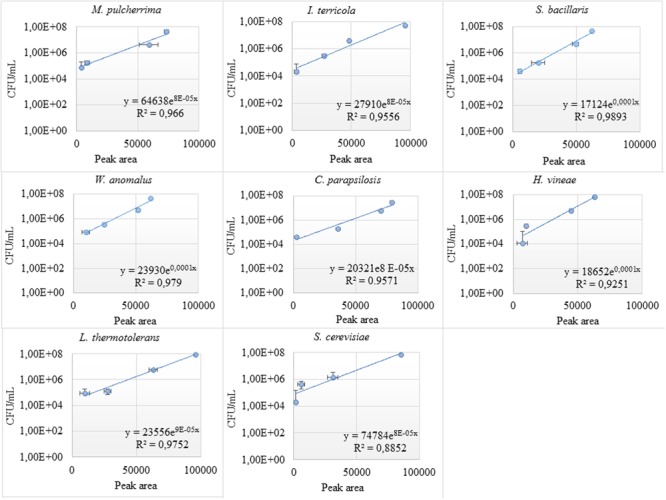
Standard curves of individual yeast species in the consortium. The correlation between the colony forming unit and peak area (bp) was investigated at different dilutions (10^3^–10^7^ CFU/mL) for individual yeast species in the consortium.

For better quantification of the individual yeast species, standard curves correlating colony forming units and peak areas were established. Strong linear correlation between CFU/mL and ARISA peak area, with an R_2_ value of ≈0.9 was observed, for individual yeast species (**Figure 3**). However, at lower biomass, the correlation between peak area and viable counts was non-linear.

### Fermentation in Synthetic Grape Juice

#### Fermentation and Growth Kinetics

**FIGURE 4:**
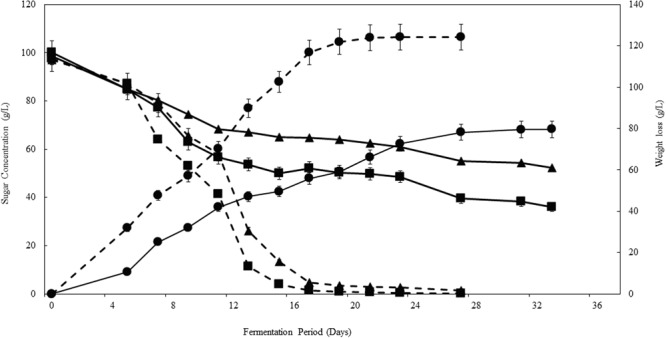
Progress curves showing the kinetics of fermentations performed in the synthetic must. Fermentation performed with *NS-Sc* consortium is indicated with broken lines while fermentation with *NS* consortium is indicated with solid lines. Glucose (■), fructose (▲) and CO_2_ release (●) were monitored throughout fermentation.

The applicability of the consortium and ARISA as a model was tested in the synthetic grape juice fermentation, inoculated with *NS-Sc* and *NS* only. The two sets of fermentations displayed distinct kinetics, with the *NS-Sc* fermentation reaching dryness (residual sugar < 2 g/L) within 21 days, while the fermentation with the *NS* consortium was sluggish and still had a total of 88 g/L residual sugar by day 30 (**Figure 4**). The *NS* fermentation got stuck at this level since the residual sugar was found to be the same after 40 days.

### Yeast Population Dynamics in Synthetic Grape Juice

**FIGURE 5:**
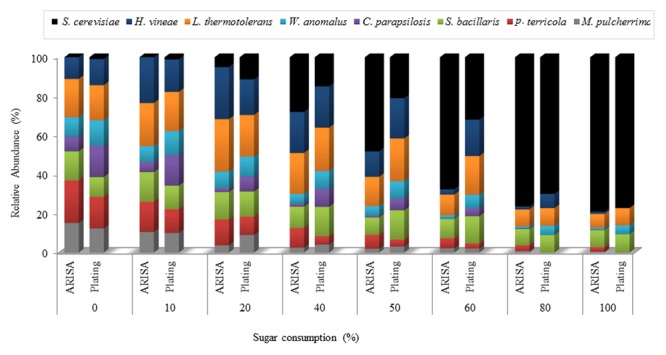
Relative abundance of yeast species throughout the *NS-Sc* fermentation in synthetic grape must. Yeast population dynamics were monitored using ARISA and plating methods.

Comparison of ARISA and viable counts from the *NS-Sc* fermentation revealed similar trends in the relative abundance of the individual species in the early stage of fermentation (**Figure 5**). However, in the middle and final fermentation stages, ARISA consistently showed higher levels of *S. cerevisiae* and lower levels of *H. vineae* than direct plating (Supplementary Table S1). In addition, *M. pulcherrima* and *P. terricola* were detectable by ARISA until the end of fermentation while, they could not be observed and enumerated on agar plates.

**FIGURE 6:**
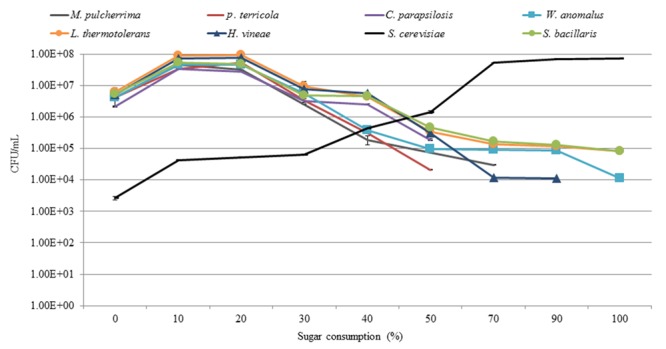
Growth profiles of yeast population throughout *NS-Sc* fermentation in the synthetic must.

Analysis of the yeast dynamics in the *NS-Sc* fermentation by standard plating on WLN agar revealed an initial increase in the population of non-*Saccharomyces* species until 10% of the sugar was consumed. The individual non-*Saccharomyces* species reached up to 10^7^–10^8^ CFU/mL and maintained viability at these levels for a brief period, before starting to decline. *P. terricola* and *C. parapsilosis*, dropped below detection by 50% sugar consumption, whereas *M. pulcherrima* and *H. vineae* were below detection after 70 and 90% sugar consumption, respectively (**Figure 6**). In contrast, the population of *S. cerevisiae* increased steadily from 10^3^ CFU/mL to 4.37 × 10^4^ CFU/mL (20% sugar consumption) where the population of all non-*Saccharomyces* species declined to 10^6^ CFU/mL. When *S. cerevisiae* reached to 6.47 × 10^4^, a decline in the population of *W. anomalus* (3.70 × 10^5^), *P. terricola* (3.10 × 10^5^) and *M. pulccherrima* (1.90 × 10^5^) was observed whereas, the population of *C. parapsolosis, H. vineae, S. bacillaris*, and *L. thermotolerans* remained at 10^6^ CFU/mL. Finally, *S. cerevisiae* dominated the fermentation and reached to 7.19 × 10^7^ CFU/mL. *L. thermotolerans* (8.40 × 10^4^), *S. bacillaris* (8.03 × 10^4^), and *W. anomalus* (1.10 × 10^4^) remained viable until the end of fermentation.

In the *NS* fermentation, the levels of *S. bacillaris, P. terricola*, and *L. thermotolerans* increased moderately and maintained dominance until 40% of the sugar was consumed while, *M. pulcherrima* and *C. parapsilosis* declined steadily from the onset of fermentation. Using the standard curves constructed as described in the previous section, the population of *S. bacillaris, P. terricola*, and *L. thermotolerans* was estimated to be 1.48 × 10^5^, 5.33 × 10^5^, and 2.82 × 10^5^ CFU/mL, respectively, whereas the population of *M. pulcherrima* and *C. parapsilosis* was 1.22 × 10^3^ and 1.69 × 10^3^ CFU/mL. The population of *H. vineae* at 40% sugar consumption was estimated to be 2.07 × 10^3^ CFU/mL.

**FIGURE 7:**
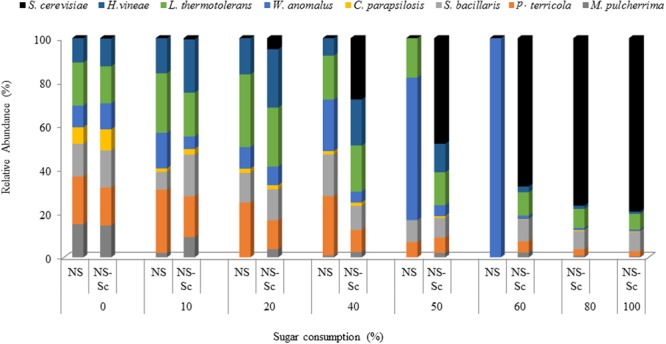
Relative abundance of yeast species during fermentations performed with *NS-Sc* and *NS*. Yeast population dynamics was monitored using ARISA.

After 50% of the sugar was consumed, only four species (*L. thermotolerans, S. bacillaris, P. terricola*, and *W. anomalus*) were detected, with *W. anomalus*, accounting for 65% of the population. The population of *L. thermotolerans, S. bacillaris, P. terricola*, and *W. anomalus* based on the standard curves were 2.74 × 10^5^, 5.58 × 10^4^, 2.77 × 10^4^, and 7.23 × 10^6^ CFU/mL, respectively. The fermentation got stuck at 60% of sugar consumption and *W. anomalus* was the only detectable yeast at this stage (**Figure 7**). The level of *W. anomalus* based on the standard curve was estimated to be 9.67 × 10^6^ CFU/mL by 60% of sugar consumption in *NS* fermentation while *S. cerevisiae* reached up to 7.19 × 10^7^ CFU/mL by the end of the *NS-Sc* fermentation.

### Chemical Parameters and Yeast Diversity in Chenin Blanc Juice

The Chenin blanc juice used in the current study was at 21.7 °Brix with a total acidity of 3.23 g/L, pH 3.37 and a yeast assimilable nitrogen (YAN) of 195 mg/L. Sugar content and YAN concentration were higher in Chenin blanc juice compared to the synthetic must (**Table 3**). One hundred and eighty four yeast isolates obtained from the Chenin blanc juice were identified and revealed that the initial indigenous yeast population comprised *M. pulcherrima* (2.39 × 10^3^ CFU/mL), *H. uvarum* (4.21 × 10^3^ CFU/mL), *L. thermotolerans* (2.70 × 10^3^ CFU/mL), *W. anomalus* (3.34 × 10^3^ CFU/mL) and *S. cerevisiae* (4.85 × 10^3^ CFU/mL).

**Table 3:** Chemical parameters of Chenin blanc compared to the synthetic grape juice.

Chemical parameter	Chenin blanc juice	Synthetic grape juice
Sugar (°Brix)	21.7	20
YAN (mg/L)	195	300
pH	3.37	3.5

### Chenin Blanc Fermentations

**FIGURE 8:**
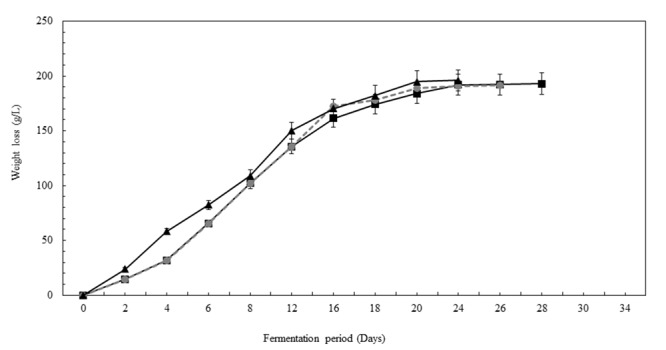
Progress curves displaying the kinetics of spontaneous fermentation (●), fermentation inoculated with *Sc* (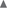), and fermentation inoculated with *NS-Sc* consortium (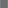).

A comparison of the spontaneous fermentation, the *Sc*-inoculated, and the *NS-Sc* inoculated fermentations, revealed that the *Sc* fermentation was the fastest and reached dryness in 24 days, followed by the spontaneous fermentation at 26 days, while, *NS-Sc* fermentation took 28 days to reach dryness (**Figure 8**).

**FIGURE 9:**
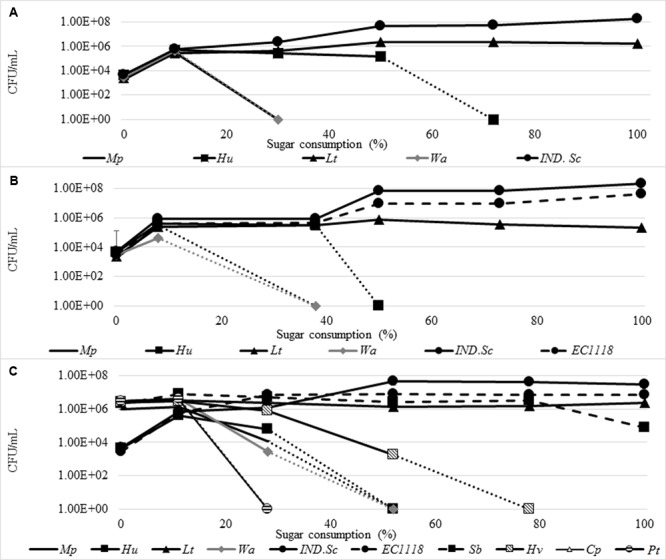
Yeast population dynamics in Chenin blanc spontaneous fermentation **(A)**, *S. cerevisiae* inoculated fermentation **(B)** and *NS-Sc* consortium fermentation **(C)**. The following abbreviations were used for names of yeast species. *Mp, M. pulcherrima; It, P. terricola; Sb, S. bacillaris; Cp, C. parapsilosis; Lt, L. thermotolerans; Hv, H. vineae; Hu, H. uvarum; IND.Sc*, Indigenous *S. cerevisiae.*

The spontaneous fermentation of the juice was characterized by an initial increase in the yeast population from ≈10^3^ CFU/mL to 6.27 × 10^5^ CFU/mL, by 10% sugar consumption. Subsequently, a decline in some non-*Saccharomyces* species was observed; amongst them, *W. anomalus* and *M. pulcherrima* declined rapidly and could not be detected by 30% sugar consumption, while *H. uvarum* persisted until 50% of the sugar was consumed. In contrast, *L. thermotolerans* increased in growth up to 2.3 × 10^6^ CFU/mL at 50% sugar consumption and persisted until the end of fermentation. The indigenous *S. cerevisiae* (*IND-Sc*) increased from ≈10^3^CFU/mL to a maximum of 1.82 × 10^8^ CFU/mL (**Figure 9A**). Similar trends were observed in the *Sc*-inoculated fermentation. However, *W. anomalus* only grew up to 4 × 10^4^ CFU/mL and *H. uvarum* persisted until 40% sugar consumption (**Figure 9B**). In addition, *L. thermotolerans* only reached a maximum of 8 × 10^5^ CFU/mL. Within the *S. cerevisiae* population, *IND-Sc* and EC1118 displayed similar growth patterns. However, *IND-Sc* persisted at a higher level, reaching a maximum of 2.1 × 10^8^ CFU/mL, while EC1118 reached 4.5 × 10^7^ CFU/mL (**Figure 9B**). When the *NS-Sc* consortium was inoculated, *H. uvarum* (the only indigenous non-*Saccharomyces* yeast that was not part of the consortium), grew from 4.4 × 10^3^ to 6.20 × 10^4^ CFU/mL by 10% sugar consumption followed by a steady decline until it could not be detected by 50% sugar consumption (**Figure 9C**). Amongst the remainder of the non-*Saccharomyces* yeasts which were inoculated at ≈10^6^ CFU/mL, *P. terricola* and *C. parapsilosis* declined below detection after 10% sugar consumption, followed by *M. pulcherrima* and *W. anomalus* by 28% sugar consumption. In contrast, *H. vineae* declined gradually until 78% sugar consumption; *S. bacillaris* persisted at 10^6^ CFU/mL until 78% sugar consumption before dropping to 8 × 10^4^ CFU/mL at the end of fermentation, while, *L. thermotolerans* persisted at 10^6^ CFU/mL until the end of fermentation. The *S. cerevisiae* population behaved in a similar way as observed in the *S. cerevisiae* inoculated fermentation, albeit at 10 times less cell concentrations. For instance, *IND-Sc* reached a maximum of 3.2 × 10^7^ CFU/mL, while EC1118 reached 6.9 × 10^6^ CFU/mL.

## Discussion

The current study aimed to establish and validate a model system for reliable monitoring and prediction of the temporal trajectories of yeast populations within the wine fermentation ecosystem. To this end, a yeast consortium comprising *S. cerevisiae* and seven non-*Saccharomyces* yeast species of varying fermentative capacities was constructed. These yeast species are all regularly encountered in SA grape juices, and some species have sometimes been detected in significant numbers. Furthermore, all of these non-*Saccharomyces* species have been isolated in countries with several wine producing regions such as Italy, France, Argentina, China, and Brazil (Jolly et al., 2003a; Combina et al., 2005; Di Maro et al., 2007; Lopandic et al., 2008; Romancino et al., 2008; Salinas et al., 2009; Sun et al., 2009; Suzzi et al., 2012; Tofalo et al., 2012; Weightman, 2014; Maturano et al., 2015; Morgan, 2016). These yeast species also differed in their ITS1-5.8S rRNA-ITS2 gene sizes, which made ARISA a suitable method to monitor their dynamics. Our data show that in this semi-complex consortium, the detection limit of ARISA could be as low as 10^3^ CFU/mL when all species are present at low levels. However, at lower biomass (10^3-4^ CFU/mL) larger deviations were observed, possibly due to the bias introduced by DNA extraction or preferential amplification in PCR (Giraffa, 2004; Ramette, 2009). Furthermore, in a typical wine fermentation scenario where dominant taxa grow up to 10^7-8^ CFU/mL, minor taxa would not be detected below 10^4^ CFU/mL. ARISA is also unable to differentiate between strains of the same species, limiting its ability to monitor strain-specific dynamics. However, species-specific interactions of significantly contributing species can be easily detected and quantified (Ramette, 2009; Ženišová et al., 2014; Ghosh et al., 2015; Setati et al., 2015). The limits are similar to those obtained for FISH (Xufre et al., 2006) and PCR-DGGE (Prakitchaiwattana et al., 2004) and they are less sensitive than qPCR (10^2^ CFU/mL) and flow cytometry (10^3^ CFU/mL) methods (Malacrinò et al., 2001; Hierro et al., 2006a,b; Zott et al., 2010). However, ARISA does not require species-specific primers and is less technically demanding than qPCR and flow cytometry. Overall, ARISA generated similar growth patterns for individual yeast species in the consortium as observed with viable counts. However, some discrepancies were observed in the middle and final stage of fermentation. These discrepancies might reflect biases and limitation in both methods. For instance, plating method might show bias against cells in a VBNC state and injured population (Divol and Lonvaud-Funel, 2005; Renouf et al., 2007) while ARISA is unable to differentiate between live and dead cells (Xie et al., 2007; O’Sullivan et al., 2013). Consequently, an overestimation of most of the species (e.g., *M. pulcherrima, P. terricola, H. vineae, L. thermotolerans, S. bacillaris, and S. cerevisiae*) by one order of magnitude was evident with ARISA compared to the plating method. The data in the current study suggest that up to 3% of dead cells could possibly be detected by ARISA. Similarly, Salinas et al., (2009) indicated that qPCR overestimate the number of live cells in average one order higher compared to microscopy analysis, which according to Hierro et al. (2006a) could represent up to 1% of the dead cells.

Our study showed that the yeast species constituting the consortium responded differently to the wine fermentation ecosystem, and the behavior of the non-*Saccharomyces* species was differentially influenced by the presence of *S. cerevisiae*. The data showed that in the absence of *S. cerevisiae*, some non-*Saccharomyces* species such as *M. pulcherrima* and *C. parapsilosis* experienced a decline from the onset of fermentation whereas, species such as *S. bacillaris, P. terricola*, and *L. thermotolerans* experienced a moderate increase followed by a steady decline in the absolute numbers by the middle of fermentation. On the contrary, *W. anomalus* suppressed the rest of non*-Saccharomyces* species and increased in cell concentration back to the initial inoculum level. This suggests that *W. anomalus* can withstand the chemical milieu created in the early stages of the fermentation better than the other yeast species and may utilize the nitrogen released by dead cells. In contrast, in the presence of *S. cerevisiae*, specifically, this yeast declines early in fermentation, suggesting that *S. cerevisiae* creates an unconducive environment, which suppresses *W. anomalus*. Indeed, an antagonistic interaction between *S. cerevisiae* and *W. anomalus*, has been proposed in other fermentation ecosystems (Ye et al., 2014). *S. cerevisiae* may inhibit other organisms through a variety of mechanisms including the production of short chain fatty acids and glycoproteins (killer toxin), and the specific antagonism exerted by *S. cerevisiae* modulates the ecosystem (Vannette and Fukami, 2014; Boynton and Greig, 2016). Conversely, other yeast species such as *M. pulcherrima, P. terricola*, and *C. parapsilosis* consistently declined in the early stages of the fermentation, both in the presence and in the absence of *S. cerevisiae*, suggesting that the decline could be due to another factor such as oxygen limitation. Several studies have shown that the growth and survival rate of *M. pulcherrima* and *C. parapsilosis* was markedly enhanced in aerated fermentations (Oh et al., 1998; Rossignol et al., 2009; Morales et al., 2015; Shekhawat et al., 2017). Furthermore, in the presence of *S. cerevisiae, L. thermotolerans*, and *S. bacillaris* could survive until late fermentation. The survival of *L. thermotolerans* until end of the fermentation has been shown previously (Gobbi et al., 2013). In addition, *S. bacillaris* strains are typically fructophilic and therefore preferentially utilize fructose, which is less preferred by *S. cerevisiae*. Interestingly, our study revealed that *H. vineae* survives better in the presence *S. cerevisiae* suggesting a positive interaction between the two yeasts. Such an interaction is perhaps not coincidental since other studies have shown that in nutrient-rich conditions, co-fermentations using strains of these two species often reflect a significant contribution of *H. vineae* to wine aroma and flavor (Viana et al., 2011; Medina et al., 2013).

Based on our current findings, we can infer that the mutualism (*S. cerevisiae* and *H. vineae*) and antagonism (*S. cerevisiae* and *W. anomalus*) observed in the wine ecosystem, could be a species-specific interaction that occurs as a result of the presence of *S. cerevisiae*. However, the strength of the mutualism or antagonism in the wine consortium may vary between different strains of one species requires further investigation. Indeed, species-specific patterns throughout the wine fermentation process are probable and comprehensible. For instance, it is well established that some species decline rapidly by early or mid-fermentation (*Cryptococcus carnescens, Aureobasidium pullulans, P. terricola*, and *M. pulcherrima)*, others repeatedly persist until late fermentation (*S. bacillaris, L. thermotolerans, T. delbrueckii*) regardless of the strain variability (Jemec et al., 2001; Sun et al., 2009; Cordero-Bueso et al., 2011; Bezerra-Bussoli et al., 2013; Gobbi et al., 2013; Milanović et al., 2013; Bagheri et al., 2015).

One of the goals of the current study was to establish a consortium that would serve as a representative model to predict yeast dynamics in wine fermentation. In order to validate the suitability of this consortium, it was used as an inoculum in Chenin blanc must and the dynamics was monitored throughout the fermentation. Interestingly, four of the yeast species (*M. pulcherrima, L. thermotolerans, W. anomalus*, and *S. cerevisiae*) which form part of the consortium were also present in the natural yeast community of the Chenin blanc must, confirming once more the representative nature of our consortium. Our study shows that all the species in the consortium could compete with the native yeast species in a non-sterilized must. While we were unable to differentiate between the indigenous strains and inoculated strains (e.g., *W. anomalus*), the population dynamics observed were similar to those described for the synthetic grape juice, suggesting species, and not strain specific drivers of interactions. This is further supported by the fact that the dynamics were preserved although the environmental conditions, including nitrogen and sugar levels, differed consoderably between the two matrices (Supplementary Table S2). We also observed that the indigenous *S. cerevisiae* population displayed better growth than the EC1118 inoculated strain although they were at similar levels at the beginning of the fermentation, further indicating that the selective drivers were species and not strain-dependent. Our data show that the consortium constructed in the current study serves as a viable and robust model to assess yeast population dynamics during wine fermentation since the matrix did not have a considerable influence on the dynamics as such. We suggest that the yeast dynamics observed in the current study is mainly due to species-specific interactions and the selective pressure applied by *S. cerevisiae* to other species. Our data suggest that inoculation with *S. cerevisiae* favors the persistence of some non-*Saccharomyces* species in wine fermentation whereas; it clearly suppresses the growth and contribution of other non-*Saccharomyces* species.

The dynamics of the wine ecosystem is driven by a multitude of positive and negative yeast–yeast interactions. The main challenge in microbial ecology is to link microbial composition to function. Here, we demonstrate that a model consortium approach can be used as a tool to predict the microbial behavior in a complex natural environment. Such a model consortium can be easily perturbed under well-controlled conditions in order to gain a deep understanding of the effect of environmental parameters on yeast–yeast interactions. In-depth insight on yeast–yeast interactions may allow us to manipulate the microbial community and enhance the population of the beneficial microbes or suppress the population of undesirable yeast species. The study presents a first step in the development of a model to predict the oenological potential of any given wine mycobiome.

## Author Contributions

FB and MS conceptualized the study. BB, FB, and MS designed the experimental layout. BB performed the experiments, analyzed the data and wrote the first draft of the manuscript. BB, FB, and MS edited subsequent drafts, read and approved the final manuscript.

## Conflict of Interest Statement

The authors declare that the research was conducted in the absence of any commercial or financial relationships that could be construed as a potential conflict of interest.
