# Supplementary material for: The Impact of Saccharomyces cerevisiae on a Wine Yeast Consortium in Natural and Inoculated Fermentations
Source: Front Microbiol. 2017 Oct 16;8:1988. doi: 10.3389/fmicb.2017.01988 (PMC5650610; doi:10.3389/fmicb.2017.01988)
Supplement: Supplementary file 1 [file Table_1.docx]

**Table S1.** Comparison between the actual viable counts as determined by plating (PL) and the total counts calculated based on ARISA peak area standard curves (STC).

| Species | CFU/mL  PL | CFU/mL  STC | CFU/mL  PL | CFU/mL  STC | CFU/mL  PL | CFU/mL  STC |
| --- | --- | --- | --- | --- | --- | --- |
|  | 0% Sugar | | 50% Sugar | | 100% Sugar | |
| *M. pulcherrima* | (42.7 ±1.56) E+05 | (15.8 ± 1.01) E+06 | (73.3 ±2.03) E+03 | (31.3 ± 3.52) E+03 | 0 | (22.4 ±1.12) E+03 |
| *P. terricola* | (41.0 ±2.85) E+05 | (18.7 ±2.41) E+05 | (21.1± 2.43) E+03 | (47 ±  3.85) E+03 | 0 | (12.5 ± 1.14) E+03 |
| *S. bacillaris* | (52.7 ±3.06)  E+05 | (44.9 ±2.47) E+06 | (45.7±3.06) E+04 | (35.1± 1.52) E+03 | (80 ± 2.74) E+03 | (15.9 ± 1.85) E+03 |
| *C. parapsilosis* | (22 ± 1.2) E+05 | (22.5 ± 2.74) E+04 | (19± 1.18) E+04 | (83.8 ± 3.17) E+03 | 0 | (73.5 ± 4.41) E+02 |
| *W. anomalus* | (42.7 ±1.03) E+05 | (25.9 ± 2.03) E+04 | (96.7± 2.31) E+04 | (12 ±2.85) E+03 | (11 ±1.65) E+03 | (22.4 ± 2.44) E+02 |
| *L. thermotolerans* | (61.7±2.08) E+05 | (50.1 ± 2.31) E+06 | (35±1.29) E+04 | (14.1 ± 2.17) E+04 | (84 ± 3.74) E+03 | (19.2± 1.17) E+03 |
| *H. vineae* | (51 ± 1.61) E+05 | (21.5 ±2.17) E+06 | (31.6 ±1.37) E+04 | (16.9± 2.07) E+05 | 0 | (82 ± 2.92) E+02 |
| *S. cerevisiae* | (26.7 ±1.51) E+02 | (25.5 ±1.81) E+03 | (14.4 ±2.41) E+06 | (83.6± 4.32) E+06 | (71.9 ± 2.6) E+06 | (11.3 ± 1.74) E+07 |
