# Supplementary material for: The Impact of Saccharomyces cerevisiae on a Wine Yeast Consortium in Natural and Inoculated Fermentations
Source: Front Microbiol. 2017 Oct 16;8:1988. doi: 10.3389/fmicb.2017.01988 (PMC5650610; doi:10.3389/fmicb.2017.01988)
Supplement: Supplementary file 2 [file Table_2.docx]

**Table S2.** Comparison between the yeast population (CFU/mL) in synthetic must and Chenin blanc must at the time of inoculation (T_INC_) and in the end of fermentation (T_EF_).

| Yeast Sp  p. | Yeast population  T_INC_ (CFU/mL)  synthetic must  (*NS-Sc*) | Yeast population  T_EF_ (CFU/mL)  synthetic must (*NS-Sc*) | Yeast population T_INC_ (CFU/mL)  Chenin blanc must (*NS-Sc*) | Yeast population  T_EF_ (CFU/mL)  Chenin blanc must (*NS-Sc*) |
| --- | --- | --- | --- | --- |
| *S. cerevisiae* (EC1118) | (26.7 ± 1.51) E+02 | (71.9± 2.6) E+06 | (29+ 1.07)  E+02 | (69± 2.87)  E+05 |
| *S. cerevisiae* (*IND-Sc*) | 0 | 0 | (48± 2.41)  E+02 | (32 ± 2.17)  E+06 |
| *S. bacillaris* | (52.7 ± 3.06) E+05 | (80 ± 2.74)  E+03 | (21± 1.65)  E+05 | (80±3.14)  E+03 |
| *L. thermotolerans* | (61.7 ± 2.08) E+05 | (84 ± 3.74)  E+03 | (23 ± 2.85)  E+05 | (23±1.19)  E+05 |
| *W. anomalus* | (42.7 ± 1.03) E+05 | (11 ±1.65)  E+03 | (30 ± 1.12)  E+05 |  |
| *H. vineae* | (51± 1.61)  E+05 | 0 | (26.3± 1.01) E+05 | 0 |
| *H. uvarum* | 0 | 0 | (44± 1.14)  E+05 | 0 |
| *M. pulcherrima* | (42.7± 1.56) E+05 | 0 | (10 ± 1.65)  E+05 | 0 |
| *P. terricola* | (41±2.85)  E+05 | 0 | (31.1± 2.54) E+05 | 0 |
| *C. parapsilosis* | (22± 1.2)  E+05 | 0 | (31.2 ± 1.12) E+05 | 0 |
