# Supplementary material for: The Impact of Saccharomyces cerevisiae on a Wine Yeast Consortium in Natural and Inoculated Fermentations
Source: Front Microbiol. 2017 Oct 16;8:1988. doi: 10.3389/fmicb.2017.01988 (PMC5650610; doi:10.3389/fmicb.2017.01988)
Supplement: Supplementary file 3 [file Image_1.pdf]

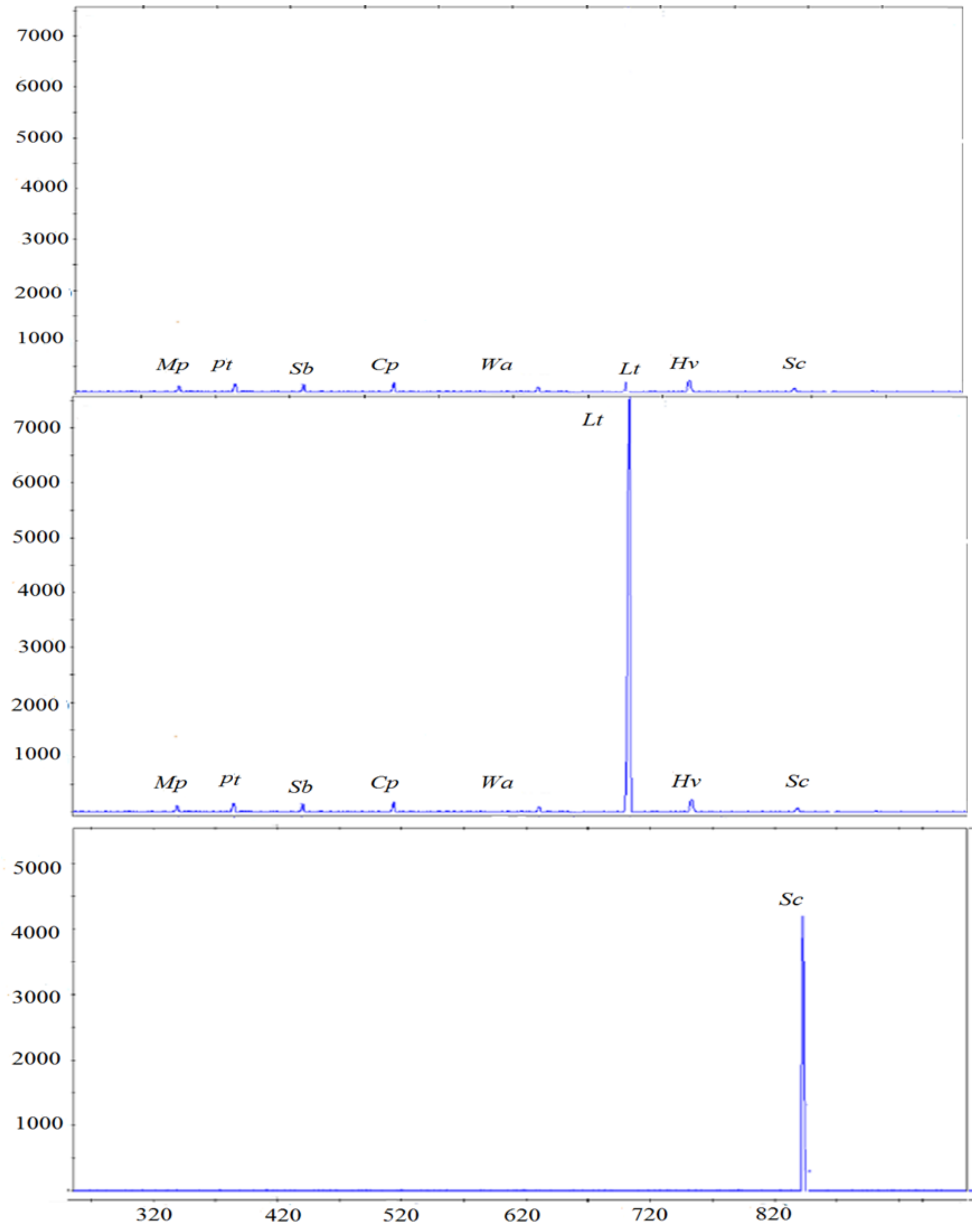

Figure S1. Electropherograms of ARISA detection limit. The x-axis represents the fragment size (bp) and the y-axis represents the relative fluorescence intensity.
