# Supplementary material for: The Impact of Saccharomyces cerevisiae on a Wine Yeast Consortium in Natural and Inoculated Fermentations
Source: Front Microbiol. 2017 Oct 16;8:1988. doi: 10.3389/fmicb.2017.01988 (PMC5650610; doi:10.3389/fmicb.2017.01988)
Supplement: Supplementary file 4 [file Image_2.pdf]

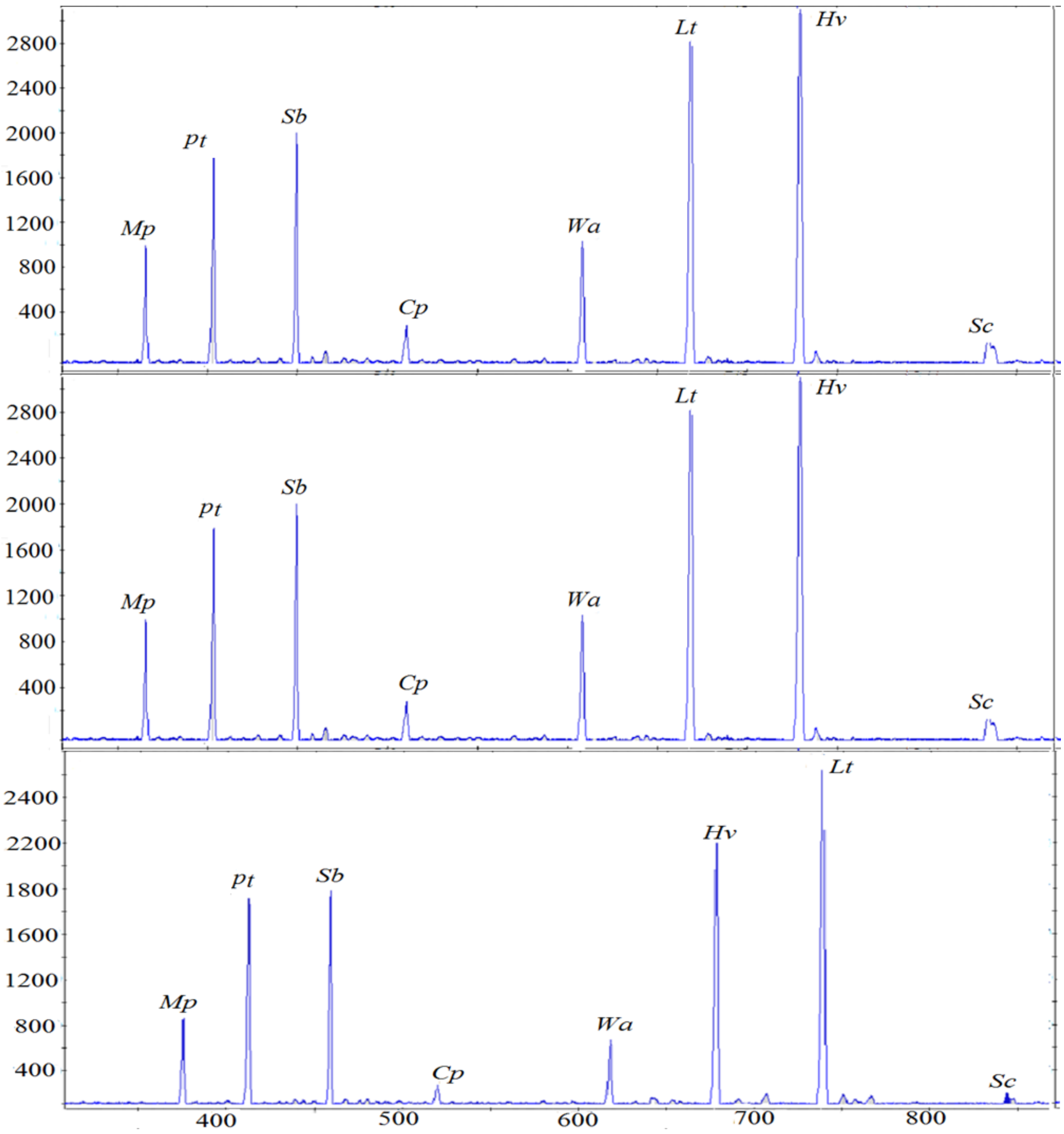

Figure S2. ARISA Electropherograms for triplicates of one sample. The x-axis represents the fragment size (bp) and the y-axis represents the relative fluorescence intensity. Three independent DNA extractions were performed for one sample. Minor variations were observed between the triplicates.
